# The use of twin-ring Ilizarov external fixator constructs: application and biomechanical proof-of principle with possible clinical indications

**DOI:** 10.1186/1749-799X-6-41

**Published:** 2011-08-11

**Authors:** Theodoros B Grivas, Evangelos A Magnissalis

**Affiliations:** 1 Orthopaedic and Trauma Department, "Tzanio" General Hospital of Piraeus, Zanni and Afendouli 1, GR-185 36, Piraeus, Greece; 2 Laboratory for the Research of the Musculoskeletal System (LRMS), University of Athens, KAT Hospital Kifissia, Athens, Greece

## Abstract

**Background:**

In peri- or intra-articular fractures of the tibia or femur, the presence of short metaphyseal bone fragments may make the application of an Ilizarov external fixator (IEF) challenging. In such cases, it may be necessary to bridge the adjacent joint in order to ensure stable fixation. The twin-ring (TR) module of circular external fixation is proposed as an alternative method that avoids joint bridging, without compromising stability of fixation. The aim of this study is to present the experimental tests performed to compare the biomechanical characteristics of the single- and TR IEF modules. The clinical application of the TR module in select patients is also presented and the merits of this technique are discussed.

**Methods:**

In this experimental study, the passive stiffness and stability of the single-ring (SR) and twin-ring (TR) IEF modules were tested under axial and shear loading conditions. In each module, two perpendicular wires on the upper surface and another two wires on the lower surface of the rings were used for fixation of the rings on plastic acetal cylinders simulating long bones.

**Results:**

In axial loading, the main outcome measure was stiffness and the SR module proved stiffer than the TR. In shear loading, the main outcome measure was stability, the TR module proving more stable than the SR.

**Discussion:**

The TR configuration, being stiffer in shear loading, may make joint bridging unnecessary when an IEF is applied. If it is still required, TR frames allow for an earlier discontinuation of bridging; either case is in favour of a successful final outcome.

**Conclusion:**

The application of the TR module has led to satisfactory clinical outcomes and should be considered as an alternative in select trauma patients treated with an IEF. Biomechanically, the TR module possesses features which enhance fracture healing and at the same time obviate the need for bridging adjacent joints, thereby significantly reducing patient morbidity.

## Background

In recent years, the use of the Ilizarov External Fixator (IEF) has been increasingly adopted for the management of complex intra- or peri-articular fractures of the knee and ankle joints. Also, the use of external fixation, based on Ilizarov principles, is invaluable in the management of difficult open tibial fractures [1].

Established indications for the application of IEF in acute trauma or the sequelae thereof include fractures in the anatomical vicinity of the knee and ankle joints, particularly those presenting with short bone fragments (e.g. a proximal tibial or a distal malleolar bone fragment), recalcitrant septic non-unions of the femur or tibia, complex fractures and non-unions of the distal femur (AO Types A1-2-3 and C1-2-3, where the distal segment is not amenable to internal fixation techniques), and cases of non- or mal-union of the foot and ankle [2-4]. Finally, the use of the IEF is also considered a viable treatment option in the management of periprosthetic distal femoral fractures (i.e. in the vicinity of a knee arthroplasty) and for elective cases, such as a proximal tibial osteotomy.

In many of the cases listed above, surgical techniques other than the IEF) are likely to compromise the soft tissue envelope (such as conventional open reduction and internal plate fixation) as well as the quality of fracture fixation (such as intramedullary nailing in a distal femur with minimal distal bone stoke as in AO Type A1), resulting in a biologically and mechanically suboptimal environment for fracture healing, [5,6].

However, the functional outcome is also highly dependent on the condition of soft tissues before treatment [1].

When applying the IEF in those cases, the surgeon often has to negotiate peri-articular bone fragments of inadequate length for placement of a sufficient number of wires with satisfactory bony purchase; if this technical difficulty cannot be overcome, the mechanical aspect of fracture fixation is compromised, risking failure of the fracture to unite.

On the other hand, one must bear in mind that there are no adverse effects of obesity, age, smoking, neuropathy, or Charcot neuroarthropathy on the complication rates and the post-operative recovery, when the IEF is used [7].

Over the past few years, our surgical team has often managed those challenging cases by incorporating a twin-ring module within the IEF frame. We observed that the final clinical outcome of this technique has been extremely rewarding in most patients. The purpose of this paper is to present the technical details associated with the application of the twin-ring (TR) IEF module in a series of select trauma patients. We also present the results of the biomechanical testing performed to investigate the properties of the proposed configuration, in comparison to a conventional, single-ring (SR) construct.

## 2. Clinical cases and outcome

### 2.1 Clinical cases

Between 2002 and 2009, our surgical team has several times used a TR module within IEF constructs, in peri- or intra-articular fractures of the femur or tibia with fragments whose short length would not allow for adequate fixation. The application of the TR IEF module proved instrumental in overcoming this technical challenge.

**Figure 1:**
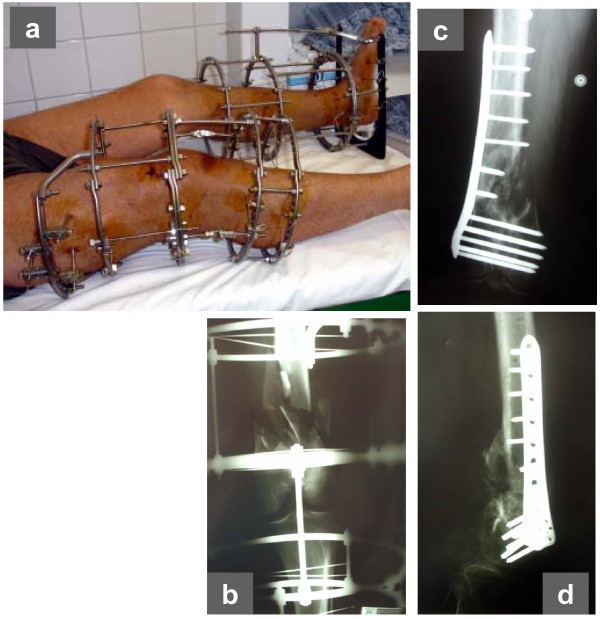
**In a multi-trauma patient treated with circular external fixator, the TR configuration was applied for the fixation of a) a right open, supracondylar femoral fracture and b) a left distal tibial (pilon) fracture (a)**. Both were high-energy injuries, resulting in severe fracture comminution and segmental bone loss from the femur at the accident site (b). Bridging of the right knee and left ankle joints was deemed necessary. Subsequently, the femoral fracture was grafted and internally fixed (c, d).

**Figure 2:**
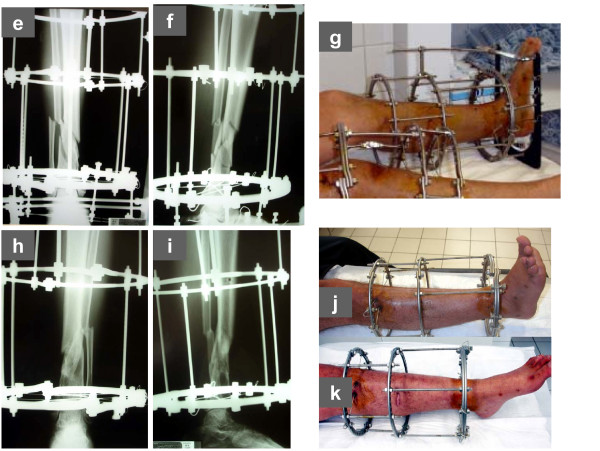
**In the patient of Figure 1, the twin-ring configuration allowed for earlier discontinuation of bridging of the ankle joint (e, f, g) during the healing period and faster joint mobilization at follow-up (h, i, j, k)**.

**Figure 3:**
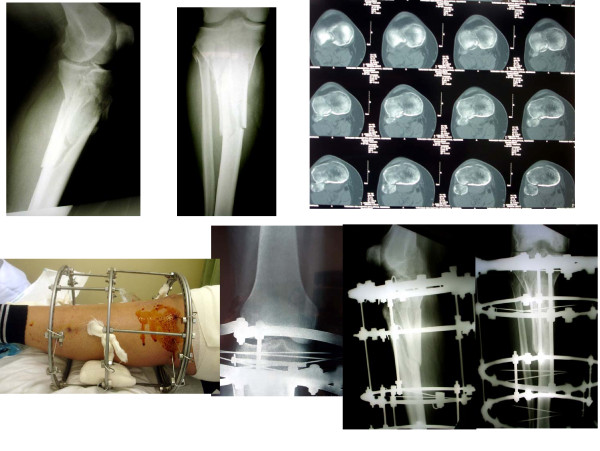
**A Schatzker type VI tibial plateau fracture treated with an Ilizarov frame featuring a twin-ring proximally in order to stabilize adequately the fracture and allow the knee joint to be mobilized as soon as possible**. The congruity of the joint line has been restored.

**Figure 4:**
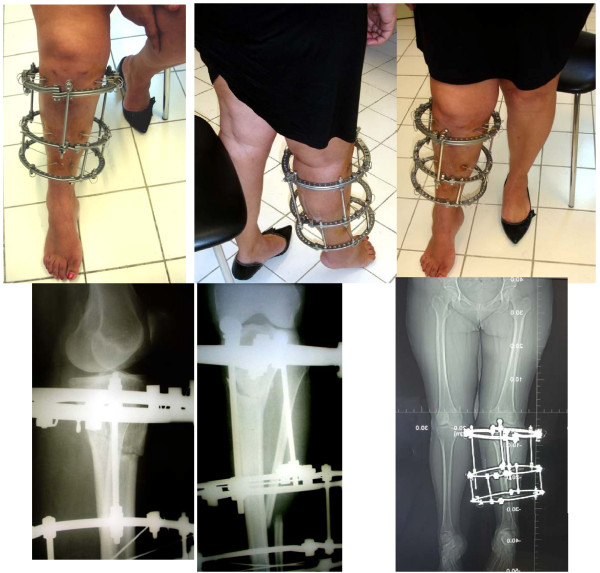
**Proximal tibial osteotomy for early-onset osteoarthritis in a 48-year-old woman**. A circular frame featuring a proximal twin-ring module was used for fixation. Stable fixation of the proximal tibial fragment with the use of the twin-ring module allowed for early mobilization of the knee joint.

**Figure 5:**
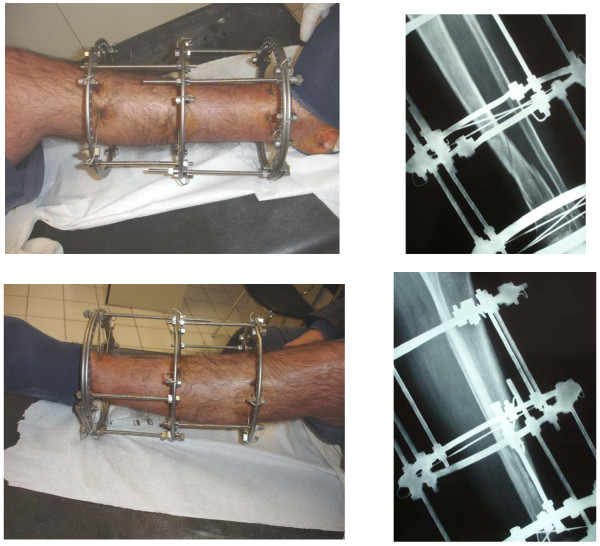
**A twin-ring module used in a circular frame for fixation of a distal tibial fracture**. The fracture line in these injuries frequently extends to the ankle joint, and the injury becomes a pilon fracture.

Table 1 outlines the indications, patient details and outcome of the use of the TR IEF (clinical cases shown in Figures 1, 2, 3, 4, 5). As shown in Table 1, the TR IEF technique proved versatile enough to manage an array of metaphyseal fractures of long bones of the lower extremities (distal femoral, proximal and distal tibial fractures, metaphyseal/epiphyseal fractures).

**Table 1:** The overall indications for use of the twin-ring IEF construct and our clinical cases and their outcome

anatomical site	type of #	cases	cases treated with IEF involving a TR module	total IEF time	adjacent joint debridging
distal femur	supracondylar # AO: A1,2,3 C1,2,3	51	8 (15.7%)	≈ 16 wks	knee: ≈ 5-6 wks earlier
	
	periprosthetic TKR #	1	1	≈ 16 wks	knee: ≈ 5-6 wks earlier
	
	as above, with delayed healing or non-union	1	1	≈ 18 wks	knee: ≈ 5-6 wks earlier

proximal tibia	condylar (plateau) # Schatzker V and VI	10	10	≈ 12 wks	knee: 4-5 wks earlier
	
	upper tibial osteotomy for management of early OA onset in young patients	1	1	≈ 13 wks	(benefit of no knee bridging)

distal tibia	supramalleolar #	10	10	≈ 16 wks	(benefit of no ankle bridging) or if the ankle will be bridged then Ankle jt debridging ≈ 3-4 wks earlier
	
	pilon #	5	5	≈ 14 wks	ankle: < 5 wks earlier
	
	pilon # with involv. of distal tibial 3rd	1	1	≈ 16 wks	ankle: < 4 wks earlier

### 2.2 Clinical outcome

The TR IEF concept demonstrated clinically relevant and ergonomically sound surgical features. The use of a TR IEF for metaphyseal/epiphyseal fractures around the knee joint or at the ankle joint provided surgeons with the option of an earlier-than-usual de-bridging of theses joints or even no bridging of the involved joint at all, thus leading to their faster mobilization of the knee or ankle joints. The overall outcome (clinical and functional) was satisfactory. There were no major postoperative complications apart the usual problem of pin site infections, that was managed with frequent wound dressing, antibiotics and infrequently with relocation of the pins.

## 3. Biomechanical testing

We hypothesized that the clinical effectiveness of the TR IEF concept might be attributed to the increased thickness of the double ring (2 × 5.0 = 10.0 mm) and the resultant increased vertical distance between its upper and lower wire levels, allowing for safe placement of up to 5 wires. As a matter of fact, this advantage was first perceived on the basis of subjective surgical judgment, providing qualitative hints to a more rigid and, therefore, mechanically sound construct. In order to validate those assumptions objectively, a series of biomechanical tests was designed, as follows.

### 3.1 Preparation of module specimens

The aim of biomechanical testing was to characterize the behaviour of single-ring (SR) vs. twin-ring (TR) IEF modules and provide proof-of-principle laboratory documentation, in a comparative manner. In order to isolate the intrinsic characteristics of the two modules, we decided to run cyclic axial and shear tests on the simplest assembly configurations possible.

The SR and TR modules consisted of two and four half-rings, respectively, all of an internal diameter of 200 mm (Cat No 10-1308). Standard Ilizarov system accessory parts (screws, washers, nuts) and 1.8 mm wires (bayonet style; Cat No 10-2102) were used to assemble the full rings and connect them to purpose-built bone-simulating models. Those were plastic acetal cylindrical parts (diameter 30 mm) meant to be loaded either axially (axial loading) or transversely (shear loading), simulating the proximal bone fragments. Precise drilling ensured appropriate insertion of Ilizarov wires at predefined transverse directions.

In both modules, four wires were used: two were drilled perpendicular to each other and were transfixed onto the upper ring surface. Another two wires were drilled at 45° to the first two and were attached on the lower surface of the ring. All ring-bolt connections were tightened to 10 Nm with an adjustable dynamometric screwdriver (model A404, Facom, France). All wires were tensioned to a level of 130 using the system's dynamometric wire tensioner (Cat No 10-3101), prior to nut tightening.

### 3.2 Testing apparatus and protocol

A materials testing machine (Imperial 2500, MECMESIN, UK) with a 1 kN load cell (ILC 1000N, MECMESIN, UK) was equipped with a device especially designed to accommodate, mount and load the SR and TR modules. This device comprised robust horizontal and vertical steel plates, as well as cannulated posts and 5 mm-thick washers used as spacers, when necessary, to negotiate step-height differences. A series of eight long M6 bolts were used to distally fix SR and TR specimens onto the device, in a safe circumferential configuration.

**Figure 6:**
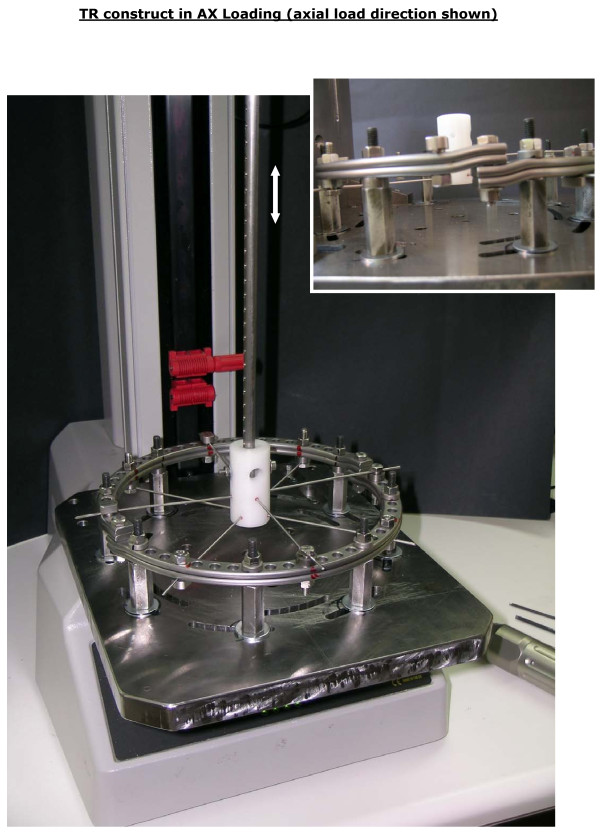
**The axial loading (AX) configuration was implemented with the plane of single- and twin-ring specimens horizontal**.

**Figure 7:**
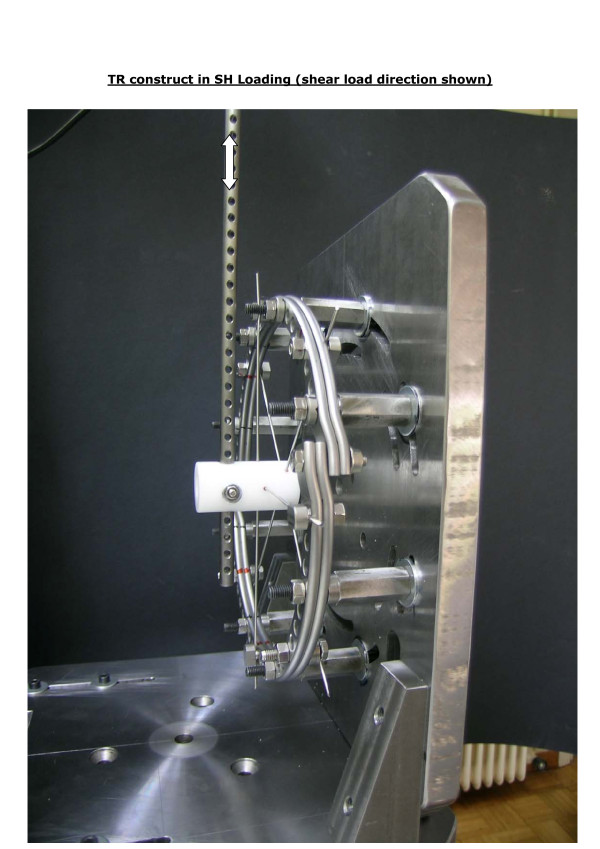
**The shear loading (SH) configuration was implemented with the plane of single- and twin-ring specimens vertical**.

A vertical displacement, input from the load cell to the bone substitute, was applied to create the required loading configurations, as follows:

- axial loading (AX) was applied with the plane of SR and TR rings horizontal (Figure 6),

- shear loading (SH) was applied with the plane of SR and TR rings vertical (Figure 7).

The load cell was equipped with a loading rod connected to the bone model through a freely pivoting pin. In both loading configurations, various cyclic loading regimens were tested (Table 2). The testing machine was always in displacement control (i.e. controlling testing speed). With a sampling frequency of 100 Hz, load, displacement and time were recorded.

**Table 2:** The tested cyclic loading regimes for Axial Loading and Shear Loading

Axial Loading		Shear Loading	
**displacement range (mm)**	**testing speed (mm/minute)**	**displacement range (mm)**	**testing speed (mm/minute)**

± 3.0	20	± 3.0	30

± 5.0	30	± 5.0	30

± 7.0	50		

### 3.3 Statistical analysis

The biomechanical differences between TR and SR were analyzed by means of a Mann-Whitney Rank Sum Test (using SigmaStat version 3.11, Systat Software, Inc.).

## 4. Results

**Figure 8:**
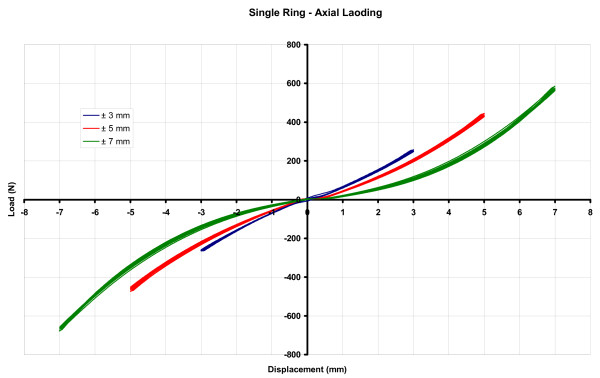
**For all axial loading tests, comparative graph of load vs. displacement**.

**Figure 9:**
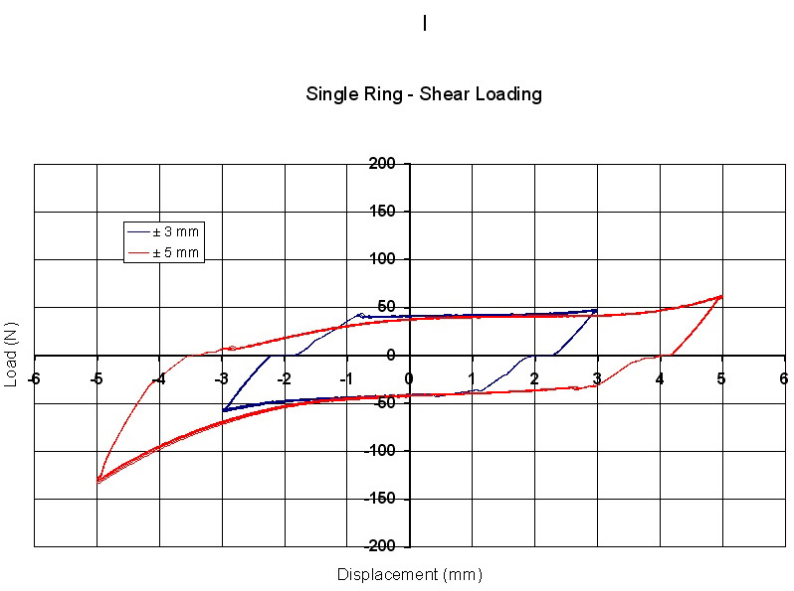
**For all shear loading tests, comparative graph of load vs. displacement**.

For all axial and shear loading tests, comparative graphs of load vs. displacement were obtained (Figures 8, 9). The effective passive stiffness of each ring module was assessed by calculating the slope values (N/mm), based on linear trends between zero and maximum displacement points (Tables 3, 4).

**Table 3:** AXIAL loading: Slope values in N/mm, characterizing the effective passive stiffness of ring modules, were calculated based on linear trends between zero and maximum displacement points

Passive Stiffness Values Average ± St.Deviation (N/mm)		AXIAL loading			
		
		negative direction	positive direction	both directions	overall
TWIN RING module	± 3 mm	67.2 ± 0.2	69.1 ± 0.2	68.2 ± 1.1	71.4 ± 3.0
		
	± 5 mm	70.2 ± 0.2	75.1 ± 0.1	72.7 ± 2.6	
		
	± 7 mm	75.3 ± 0.2	71.2 ± 0.2	73.2 ± 2.1	

SINGLE RING module	± 3 mm	92.3 ± 0.2	92.2 ± 0.4	92.2 ± 0.3	94.4 ± 2.9
		
	± 5 mm	98.0 ± 0.2	95.1 ± 0.2	96.6 ± 1.5	
		
	± 7 mm	98.0 ± 0.2	91.1 ± 0.3	94.5 ± 3.6	

**Table 4:** SHEAR loading: Slope values in N/mm, characterizing the effective passive stiffness of ring modules, were calculated based on linear trends between zero and maximum displacement points

Passive Stiffness Values Average ± St.Deviation (N/mm)		SHEAR loading			
		negative direction	positive direction	both directions	overall
TWIN RING module	± 3 mm	26.8 ± 0.1	28.9 ± 0.4	27.9 ± 1.1	27.9 ± 1.3
		
	± 5 mm	29.3 ± 0.5	26.6 ± 0.5	28.0 ± 1.5	

SINGLE RING module	± 3 mm	19.3 ± 0.6	15.9 ± 0.5	17.6 ± 1.9	18.5 ± 5.3
		
	± 5 mm	26.4 ± 0.6	12.4 ± 0.3	19.4 ± 7.4	

The mean biomechanical responses of TR and SR modules may be summarized as follows: in axial loading, the TR module demonstrated a lower stiffness than the SR module (71.4 ± 3.0 N/mm vs. 94.4 ± 2.9 N/mm, respectively), while in shear loading the stiffness of the TR was higher than that of the SR module (27.9 ± 1.3 N/mm vs. 18.5 ± 5.3 N/mm, respectively).

These biomechanical differences between TR and SR were found to be statistically significant (*p *< 0.001), both in axial and shear loading, (Mann-Whitney Rank Sum Test).

## 5. Discussion

When treating trauma patients with an IEF, the surgeon is not infrequently faced with the challenge of fractures involving relatively short-length bone fragments (for instance, in the proximal or distal tibial metaphysis). In those circumstances, it may be necessary to extend the frame beyond the involved joint in order to maximize the mechanical stability of the construct. In doing so, however, this joint is unnecessarily "locked" in place together with the fractured bone fragments. The resultant immobilisation of the articular surfaces may have detrimental implications for the final outcome as it is classically known that immobilization of the articular surfaces is associated with adverse sequelae, including stiffness or even arthritis.

As an alternative, it was thought that a twin-ring module could be used for the ring located adjacent to the short bone fragment. Our surgical team has proudly observed satisfactory clinical and functional outcomes in select trauma cases where the twin-ring module was used. It remained to be proven, however, that this configuration possessed adequate mechanical features. As a proof-of-principle procedure, we decided to run a series of biomechanical tests, in order to comparatively characterize the behaviour of single- and twin-ring IEF modules.

In axial loading, curves of load vs. displacement demonstrated a trend which was close to linear at low amplitudes (± 3 mm) and less so at higher (± 5 and ± 7 mm) amplitudes. In all cases, however, trends were continuous and quite regular, enabling us to quantify values of passive stiffness as the linear slopes between the zero and maximum displacement points. In all instances, the TR module demonstrated clearly lower values of passive stiffness than the SR module. The phenomenon of wire-pretension-loss during axial loading (long recognized and still investigated for IEF [8-12], although not detrimental in any case, is expected to be more clearly manifested in TR rather SR modules; since the increased vertical distance of TR wire levels induces successive rather than simultaneous loading of wire levels and therefore a less stiff behaviour. In the current context of TR modules, a lower stiffness in axial loading is thought to be beneficial, as it allows for the necessary axial micromotion and consequent compression between bone fragments [13].

In shear loading, curves of load vs. displacement demonstrated a trend consisting of distinct loops. The loops had a longer dimension along the x- (displacement) than along the y-axis (load). This pattern can be explained by the fact that, upon application of shear load, the plastic model simulating bone first slides against the wire aligned to the direction of load and then transfers the load to the construct. Our analysis deliberately ignored intermediate sliding events and was focused on the overall effective behavior of constructs instead. Therefore, it was conducted between zero and maximum displacement points. In all instances, the TR module demonstrated clearly higher values of passive stiffness than the SR module. A higher stiffness in shear loading is thought to be beneficial because it resists motion along the axial (transverse) plane of bone segments, potentially jeopardizing, or even disorganizing, callus formation [14].

The present biomechanical study was conceived, designed and executed in order to comparatively provide an insight to the mechanical performance of single- and double-ring modules of Ilizarov constructs. By eliminating potential confounding factors, the experimental setup and methodology ensured a comparative demonstration of biomechanical events, leading to a set of reliable findings with clinical implications.

This work may be expanded (and further research is currently under way) to encompass more elaborate mechanical testing, with different wire configurations and loading conditions (e.g. torsion). Furthermore, in an effort to create a permanent numerical model of Ilizarov constructs, computational studies involving finite element modeling and analysis (FEM-FEA) on both module configurations, can be undertaken.

The results of the present study have implications in the clinical setting. In cases of knee and ankle intra- or peri-articular fractures, it is often considered necessary to extend the IEF so as to span the involved joint, for the purposes of increased stability in bending loads. By being stiffer in shear loading, use of the twin-ring configuration may achieve an equally stable fixation without the need to bridge a joint. If a surgeon still decides to span a joint, the twin-ring module allows for earlier removal of the frame. In either case, an optimal clinical outcome is more likely. Furthermore, and possibly more importantly, in the immediate postoperative period, when the limb is immobilized for a 2-3 week period, neovessel formation occurs at the fracture region [15]. After the initiation of weight-bearing, the twin-ring system is more flexible in axial loading. This increased flexibility exploits those neovessels and promotes fracture healing, while the increased shear stiffness prevents horizontal micromotion and development of a non- or mal-union.

It is accepted that pin loosening and subsequent pin track infection [16] is a problem of rather mechanical aetiology, and usually occurs at the proximal- or distal-most ends of the external fixator.

Pin track infection also appeared in some of our cases and was treated with meticulous local cleaning and dressing, antibiotics and rarely with pin exchange. In this report this IEF treatment complication is not mentioned in detail as we principally focus on the technical aspects of TR configuration.

This complication has been attributed to local bending effects, particularly those in the vicinity of joints and can be effectively addressed either by extending the construct across the joint or by locally increasing the number of wires. Both options aim at increasing mechanical stability and enhancing bony union. The latter, however, is preferable and can be implemented more easily in a TR IEF configuration.

The TR Ilizarov construct possesses favourable biomechanical properties, which enhance fracture union and at the same time reduce complications arising from joint immobilization and pin/wire track infections. Our clinical outcomes, corroborated by the biomechanical proof-of-principle, are encouraging enough for us to recommend its continued use in patients with the appropriate clinical indications.

## 6. Conclusions

When treating select trauma patients, the application of the TR module in Ilizarov circular external fixators is suggested as a promising surgical alternative, offering satisfactory outcomes and reduced patient morbidity.

## Competing interests

The authors declare that they have no competing interests.

## Authors' contributions

TBG introduced the twin Ilizarov ring configuration, was the surgeon of all the treated patients with this TR module, described the indications of treatment, was responsible for conception and supervision of the study, planning of the experiments, and drafting the manuscript. EAM was responsible for planning of the experiments, performed the biomechanical testing and contributed with analysis of the data and drafting of the manuscript. All authors read and approved the final manuscript.
